# Patients With Ileal Pouch-Anal Anastomosis Have Decreased Bowel Frequency on Glucagon-Like Peptide-1 Receptor Agonist Therapy

**DOI:** 10.14309/ctg.0000000000001070

**Published:** 2026-07-01

**Authors:** Ezan Chaudhry, Payal Kakadiya, Hayden Vaughn, Simon Gray, Jonathan J. Hansen, John P. Haydek, Animesh Jain, Millie D. Long, Hans H. Herfarth, Edward L. Barnes

**Affiliations:** 1 School of Medicine, University of North Carolina at Chapel Hill, Chapel Hill, North Carolina, USA;; 2 Department of Pharmacy, University of North Carolina Health, Chapel Hill, North Carolina, USA;; 3 Division of Gastroenterology and Hepatology, University of North Carolina at Chapel Hill, Chapel Hill, North Carolina, USA;; 4 Multidisciplinary Center for Inflammatory Bowel Diseases, University of North Carolina at Chapel Hill, Chapel Hill, North Carolina, USA.

**Keywords:** GLP-1 receptor agonists, ileal pouch-anal anastomosis (IPAA), ulcerative colitis, frequency, pouchitis

## Abstract

**INTRODUCTION:**

High bowel frequency after ileal pouch-anal anastomosis (IPAA) causes significant symptom burden. We aimed to evaluate the impact of glucagon-like peptide-1 receptor agonists (GLP-1 RAs) in this setting.

**METHODS:**

We conducted a retrospective cohort study of patients with previous IPAA for ulcerative colitis who were treated with a GLP-1 RA (n = 20). Daily bowel frequency at baseline and 12 weeks after GLP-1 RA initiation was assessed. We also assessed the proportion of patients achieving ≥30% reduction in bowel frequency and ≤8 bowel movements per day.

**RESULTS:**

The median daily bowel frequency decreased from 9.0 (interquartile range [IQR], 6.0–12.5) at baseline to 6.0 (IQR, 5.0–8.1) at the 12-week follow-up (*P* < 0.01). Overall, 7 of 20 patients achieved a ≥30% reduction in bowel frequency, and 15 of 20 had ≤8 bowel movements per day.

**DISCUSSION:**

In this retrospective cohort, we provide further evidence for the potential role of GLP-1 RAs in the management of high bowel frequency after IPAA.

## Graphical abstract

**Figure:**
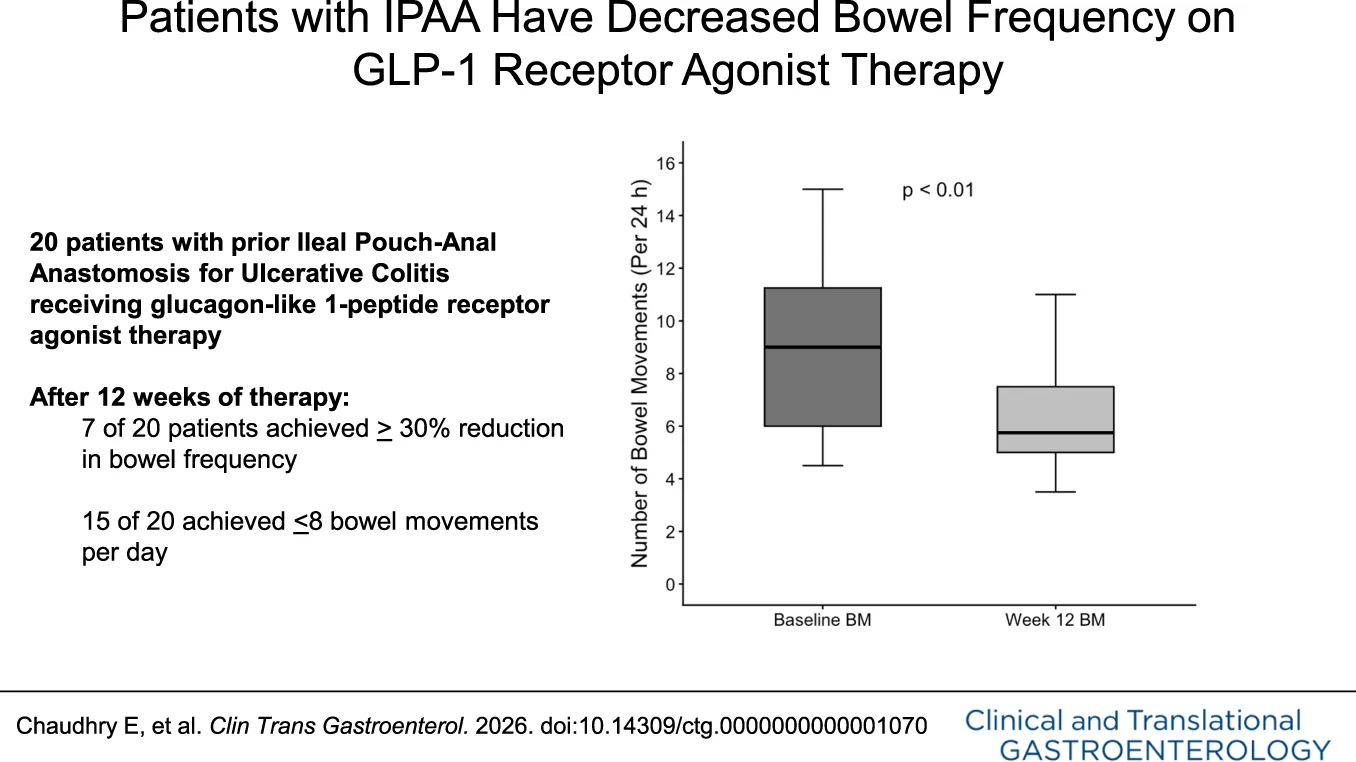

## INTRODUCTION

Although intermittent pouchitis is the most common complication after ileal pouch-anal anastomosis (IPAA) for ulcerative colitis (UC), many patients experience high bowel frequency with or without active pouch inflammation (1). This increased daytime and nocturnal bowel frequency significantly impairs quality of life (2,3).

The pathophysiology of increased bowel frequency after IPAA is multifactorial, involving dysbiosis, altered pouch motility, and potentially reduced glucagon-like peptide-1 (GLP-1) concentrations, a hormone that physiologically slows postprandial gastrointestinal transit (4–6).

In patients without active inflammation, management remains challenging, as first-line therapies such as antidiarrheal and anticholinergic medications are not effective in 54% of patients (7). GLP-1 receptor agonists (GLP-1 RAs) have emerged as potential therapies for the management of high bowel frequency after IPAA based on their effectiveness in managing high ostomy output and their modulation of the ileocolonic brake (5,8). We recently demonstrated the efficacy of the GLP-1 RA liraglutide in patients with elevated baseline bowel frequency, observing a >35% reduction in bowel movements over 24 hours. (9). Given the suggested benefit of GLP-1 RA therapy in this previous proof-of-concept study, we evaluated the impact of GLP-1 RA therapy for high bowel frequency after IPAA using multiple agents, including those with potentially more convenient dosing regimens, in a real-world setting.

## METHODS

Eligible patients were those who received an IPAA after UC and who were prescribed a GLP-1 RA in the University of North Carolina Multidisciplinary Inflammatory Bowel Disease (IBD) Clinic between September 1, 2020, and May 31, 2025. Inclusion criteria required adherence to a GLP-1 RA during the study period and availability of baseline and follow-up bowel frequency data. Clinical and demographic data were extracted from the electronic health record. Surgical and disease-related variables included indication for surgery, date of IPAA surgery, IPAA diagnosis, and history of primary sclerosing cholangitis. We evaluated the specific GLP-1 RA prescribed, as well as concomitant IBD-specific and antidiarrheal medications at GLP1RA initiation. GLP-1 RA therapy was prescribed at the clinician's discretion, and there were no inclusion criteria specific to the indication of GLP-1 RA prescription. Documentation of pouchoscopy before GLP-1 RA initiation was not required.

The primary outcome was the change in median daily bowel frequency at 12 weeks after initiation of GLP-1 RA therapy. For patients without a clinic visit at 12 weeks, stool frequency was derived from the closest documented time point. Secondary end points included the proportion of patients achieving ≥30% reduction in bowel frequency from baseline, the proportion achieving ≤8 bowel movements per day at 12 weeks, and the safety and tolerability of GLP-1 RA therapy.

Change in bowel frequency from baseline to 12 weeks was assessed using the Wilcoxon signed rank test. Categorical responder outcomes were compared using χ^2^ analysis and the Fisher exact test as appropriate. All analyses were conducted using SAS, version 9.4 (SAS Institute, Cary, NC).

## RESULTS

We identified 37 patients treated with GLP-1 RA therapy after IPAA, of whom 20 met the inclusion criteria and comprised the final analytic cohort. The median age at GLP-1 RA initiation was 55 years (interquartile range [IQR], 43.0–59.8), with a slight female predominance (55%; Table 1). More than half of patients (55%) were receiving concomitant advanced IBD therapy at GLP-1 RA initiation, and 40% were using antidiarrheal medications. Semaglutide was the most prescribed GLP-1 RA (65%).

**Table 1.** Baseline demographics, surgical, and clinical characteristics

	Patients treated with a GLP-1 RA N = 20	
	**Median**	**IQR**
Age at GLP-1 RA initiation (yr)	55.0	43.0–59.8
Body Mass Index at GLP-1 RA initiation (kg/m^2^)	30.2	27.4–35.0
Years since IPAA	13.0	8.8–16.0
	**N (%)**	**%**
Male sex	9	45
Female sex	11	55
Race		
White	16	80
Black or African American	2	10
Other	2	10
Ethnicity (Hispanic or Latino)	2	10
Indication for colectomy		
Medically refractory disease	16	80
Dysplasia or cancer	1	5
Multiple indications	2	10
Unknown	1	5
Disease extent prior to colectomy		
Proctitis	2	10
Left-sided colitis	3	15
Extensive colitis	15	75
IPAA diagnosis		
Normal	2	10
Chronic antibiotic dependent pouchitis	8	40
Chronic antibiotic refractory pouchitis	2	10
Crohn's-like disease of the pouch	8	40
IPAA stage		
One stage	4	20
Two stage	13	65
Three stage	3	15
Concomitant IBD therapy^a^	11	55
Biologics	7	35
Small molecule	1	5
Steroids	2	10
Amino salicylates	3	15
Concomitant antibiotic therapy	7	35
Concomitant antidiarrheal medications	8	40
GLP-1 RA type		
Semaglutide	13	65
Tirzepatide	1	5
Liraglutide	4	20
Dulaglutide	2	10

GLP-1 RAs, glucagon-like peptide-1 receptor agonists; IBD, inflammatory bowel disease; IPAA, ileal pouch-anal anastomosis; IQR, interquartile range.

a Patients may have been receiving more than 1 concomitant IBD therapy at the time of GLP-1 RA initiation.

**Figure 1.**
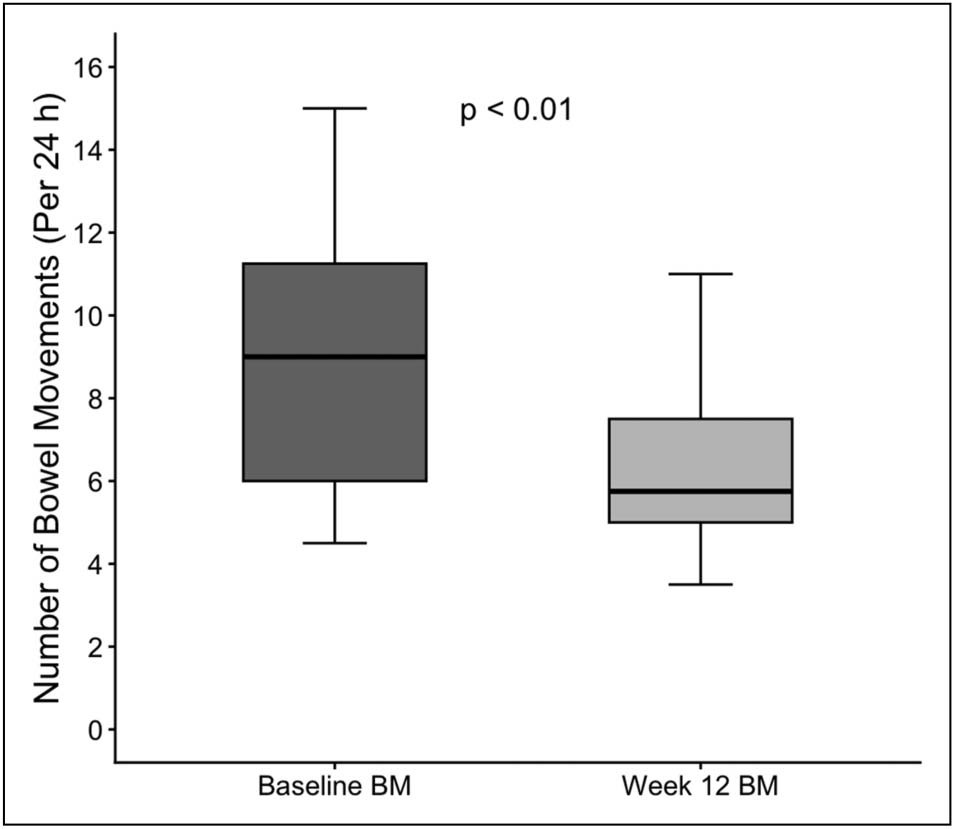
Comparison of median daily bowel frequency at baseline and week 12 among patients receiving glucagon-like peptide-1 receptor agonists.

The median daily bowel frequency decreased from 9.0 (IQR, 6.0–12.5) at baseline to 6.0 (IQR, 5.0–8.1) at 12 weeks (*P* < 0.01, Figure 1). Overall, 7 of 20 patients achieved ≥30% reduction in bowel frequency. In addition, 15 of 20 achieved ≤8 bowel movements per day at 12 weeks compared with 8 of 20 at baseline (*P* = 0.03). Responder rates did not differ between patients treated with semaglutide and those treated with other GLP-1 RAs for ≥30% reduction in bowel frequency (4 of 13 vs 3 of 7, *P* = 0.65) or achieving ≤8 bowel movements per day (9 of 13 vs 6 of 7, *P* = 0.42). There were no significant differences when comparing the likelihood of achieving a ≥30% reduction in bowel frequency or ≤8 bowel movements per day when evaluating by pouch phenotype (normal pouch, intermittent/acute pouchitis, chronic antibiotic-dependent pouchitis, chronic antibiotic-refractory pouchitis, and Crohn's-like disease of the pouch).

One patient developed persistent nausea attributed to GLP-1 RA use, which resolved after discontinuation. No other adverse events were noted during the study period. Patients lost a median of 4.7 kg (IQR 1.6–7.5) in the 12 weeks after GLP-1 RA initiation, representing a median change in total body weight of -5.98%.

## DISCUSSION

We demonstrated a significant reduction in bowel frequency among patients after IPAA for UC. Given that bowel frequency is among the most problematic symptoms after IPAA, identifying novel therapeutic approaches is important. In confirming the success of GLP-1 RA therapy previously demonstrated in a proof-of-concept trial, we expand the evidence supporting their use in this real-world setting and offer alternative approaches to the standard anticholinergic and antidiarrheal medications that have served as the foundation of therapy to date.

The observed reduction in bowel frequency with GLP-1 RA therapy is biologically plausible, given the established effects of GLP-1 on gastrointestinal motility and neuroendocrine regulation. In patients who undergo total proctocolectomy with IPAA, the removal of the colon results in loss of colonic enteroendocrine L cells, a major source of endogenous GLP-1. This disruption may attenuate the GLP-1-mediated inhibition of gastrointestinal motility, potentially leading to rapid intestinal transit and increased bowel frequency (5,6,8). Exogenous GLP-1 RA administration may partially restore this brake mechanism, thereby slowing transit and reducing stool frequency. The suggested impact of GLP-1 RA in this cohort, where patients had significant symptom burden despite multiple concomitant therapies at baseline, indicates the potential benefit of GLP-1 RA after IPAA.

Although our findings suggest potential benefit, several limitations warrant consideration. The retrospective design precludes causal inference, and the small sample size in this single-center cohort limits the ability to adjust for potential confounders. Variability in clinical practice contributed to heterogeneity in GLP-1 RA selection, dosing, titration, and follow-up timing, precluding assessment of dose-response relationships and potentially introducing variability in treatment effects. Specifically, we cannot assume or accurately assess the indication for GLP-1 RA in this retrospective cohort. In addition, bowel frequency outcomes were derived from clinically documented patient reports rather than standardized diaries or validated patient-reported outcome instruments. There is the potential that GLP-1 RA affects outcomes through anti-inflammatory mechanisms as well (10); however, in this short-term follow-up period, we did not have objective data such as calprotectin or repeated pouchoscopy assessments to evaluate these associations.

In summary, GLP-1 RA therapy may represent a promising adjunctive therapeutic option for managing high bowel frequency after IPAA. Given the limited medical therapies available for symptom control in this population, GLP-1 RAs may offer a mechanistically targeted approach to modulating gastrointestinal motility. Future prospective studies with standardized dosing, structured outcome assessment, and longer follow-up are needed to further define their therapeutic role.

## CONFLICTS OF INTEREST

**Guarantor of the article:** Edward L. Barnes, MD, MPH, FACG.

**Specific author contributions:** All authors contributed to the study concept and design, acquisition of data, interpretation of results, and critical revision of the manuscript. E.C. and E.L.B. conduced the statistical analysis and drafted the manuscript. P.K. also contributed to the drafting of the manuscript.

**Financial support:** This research was supported by grants from the National Institutes of Health [K23DK127157 (ELB)].

**Potential competing interests:** Millie D. Long has served as a consultant for AbbVie, Celltrion, Eli Lilly, Intercept, Takeda, Johnson and Johnson, Pfizer, Sanofi, Target RWE and has received research support from Pfizer, Eli Lilly, Celltrion and Takeda. Hans H. Herfarth has served as a consultant for Boxer Capital, Celltrion, Evommune, ExeGI, Fish&Richardson, Gilead, ICON, Immundiagnostik, Janssen, Lilly, Pfizer, TEVA, Ventyx and has received research support: Lilly, Novo Nordisk, and Pfizer. Edward L. Barnes has served as a consultant for AbbVie, Boomerang, Eli Lilly, Pfizer, Johnson & Johnson, Pfizer, Sanofi, Takeda, and Target RWE. He has research support from Eli Lilly and Bausch Health/Salix. Payal Kakadiya, Hayden Vaughn, Simon Gray, Jonathan Hansen, John P. Haydek, Animesh Jain have no relevant conflicts of interest.

**IRB statement:** This study was approved by the University of North Carolina Institutional Review Board (25–1717).

## ABBREVIATIONS:

BM: bowel movements
GLP-1 RAs: glucagon-like peptide-1 receptor agonists
GLP-1: glucagon-like peptide-1
IBD: inflammatory bowel disease
IPAA: ileal pouch-anal anastomosis
IQR: interquartile range
UC: ulcerative colitis
UNC: University of North Carolina
